# Altered auditory processes pattern predicts cognitive decline in older adults: different modalities with aging

**DOI:** 10.3389/fnagi.2023.1230939

**Published:** 2023-09-06

**Authors:** Junjie Yang, Xiaochen Tang, Shaohui Lin, Lijuan Jiang, Kai Wei, Xinyi Cao, Lingshan Wan, Jijun Wang, Hansheng Ding, Chunbo Li

**Affiliations:** ^1^Shanghai Key Laboratory of Psychotic Disorders, Shanghai Mental Health Center, Shanghai Jiao Tong University School of Medicine, Shanghai, China; ^2^Shanghai Health Development Research Center, Medical Information Center, Shanghai, China; ^3^Institute of Psychology and Behavioral Science, Shanghai Jiao Tong University, Shanghai, China; ^4^CAS Center for Excellence in Brain Science and Intelligence Technology (CEBSIT), Chinese Academy of Science, Shanghai, China

**Keywords:** sensory impairment, auditory discrimination, mismatch negativity (MMN), cognitive decline, brain aging

## Abstract

**Background:**

Cohort studies have shown that older adults with hearing impairment as assessed by self-report or behavioral measures are at higher risk of developing dementia many years later. A fine-grained examination of auditory processing holds promise for more effective screening of older adults at risk of cognitive decline. The auditory mismatch negativity (MMN) measure enables one to gain insights into the neurobiological substrate of central auditory processing. We hypothesized that older adults showing compromised indexes of MMN at baseline would exhibit cognitive decline at the one-year follow-up.

**Methods:**

We performed cognitive evaluations with the Repeatable Battery for the Assessment of Neuropsychological Status (RBANS; Form A and Form B) in 108 community-dwelling older adults and acquired EEG via the classic passive auditory oddball paradigm at baseline and 12-month follow-up.

**Results:**

The results showed that young-old adults with future cognitive decline showed a decrease in MMN peak amplitude, accompanied by a forward-shifting latency, whereas in older adults it showed a delay in MMN latency, and unchanged MMN peak amplitude at midline electrodes (Fz, FCz and Cz). Furthermore, the peak amplitude of the MMN decreases with age in older adults aged 70–80 years rather than 60–70 years or > 80 years.

**Conclusion:**

The altered MMN model exists in different aging stages and it’s a promising electrophysiological predictor of cognitive decline in older adults. In addition, further research is needed to determine the neural mechanisms and potential implications of the accelerated decline in MMN in older adults.

## Introduction

Sensory impairment, especially hearing and visual impairments may be potentially modifiable risk factors, as they are individually associated with increased risk for dementia (Maharani et al., 2018; Livingston et al., 2020; Maharani et al., 2020). Hearing impairment is a stronger risk factor for dementia than visual impairment and has a more wide-ranging effect on different subtypes of dementia (Fischer et al., 2016). Current studies on the relationship between cognition and sensory impairment have detected sensory impairment in older adults through self-report or behavioral measurements (Maharani et al., 2020). The causal relationship between hearing and cognitive impairment and the possible neural mechanisms is unclear. Recent animal experiments on rats and gorillas have found the effects of auditory processing deficit on the brain are widespread involving the frontal cortex as well as hippocampus-related structures by compromising white matter integrity and disrupting neuronal plasticity, and leading to cognitive impairment, unlike the visual processing deficit (Gray et al., 2020; Zhang et al., 2021). Studies suggest that older adults show impaired supra-threshold auditory processes (which cannot be identified by a clinical audiogram), and the association between hearing impairment and cognitive impairment may be present at earlier stages (Plack et al., 2014; Golub et al., 2020). Auditory processing may provide a window for detecting individuals at high risk of cognitive decline in older adults and for further developing effective interventions.

The auditory mismatch negativity (MMN) measure enables one to gain insights into the neurobiological substrate of central auditory processing and various attention-related processes (Naatanen et al., 2014). The MMN response is seen as a negative displacement in particular at the frontocentral and central scalp electrodes (relative to a mastoid or nose reference electrode) in the difference wave obtained by subtracting the event-related potential (ERP) to frequent, “standard,” stimuli from that to deviant stimuli. The MMN usually peaks at 150–250 ms from change onset, with this peak latency getting shorter with the increasing magnitude of stimulus change (Naatanen et al., 2007). The trend of decreasing MMN peak amplitude with increasing age in adulthood is well established, but the evidence is sparse for older people >70 years (Kiang et al., 2009; Cheng et al., 2013). We were interested in checking whether auditory MMN could be used as a predictor of the risk of cognitive decline in older adults, and the possible effects of age. We hypothesized that older adults showing compromised indexes of MMN at baseline would exhibit cognitive decline later.

## Methods

### Study participants

One hundred eight community-dwelling older adults were recruited by advertisements participated in this study. Eligibility criteria were as follow: no difficulties with hearing, vision, or communication; no severe physical or psychotic disorder; and no obvious cognitive decline [the Chinese version of the Mini-Mental State Examination (MMSE) ≥19 for elementary education and ≥ 24 for middle school education and above; the normal cutoff point of the MMSE score is lower because of the lower educational level in China (Li et al., 2006)]. Exclusion criteria included the following: obvious cognitive decline such as Alzheimer’s disease (AD); history or clinical evidence of neurological disease or psychiatric disorder such as brain cancer, major depressive disorder, or schizophrenia. The experimental protocol was approved by the Ethics Committee in Shanghai Mental Health Center (SMHC, approval No 2018-06) in compliance with the Helsinki Declaration. Written informed consent was obtained from each participant before the experiments. Each participant was compensated for their participation. Two individuals were excluded from the analysis, one due to inadequate quality of EEG data and the other due to abnormal cognitive scores outside the mean + 3SD range.

### Measurements

#### Assessment of cognitive function

Cognitive measurements were conducted at baseline and the 12-month follow-up. Immediate memory (list learning and story memory), visuospatial/constructional (figure copy and line orientation), language (picture naming and semantic fluency), attention (digit span and coding), and delayed memory (list recall, list recognition, story recall, and figure recall) index scores were obtained from the Repeatable Battery for the Assessment of Neuropsychological Status (RBANS; Form A and Form B), which has been verified in terms of its good reliability and validity in Chinese community-dwelling older adults (Cheng et al., 2011).

#### Covariates

Baseline covariates include education, gender, self-rating anxiety scale (SAS) score and self-rating depression scale (SDS). For demographic profiles, 6 participants have missing years of education and 5 participants have missing SAS and SDS scores.

#### Auditory oddball paradigm

All 108 participants completed EEG acquisition at baseline. Auditory stimuli consisted of one standard tone (1 kHz, 50 ms) and two deviants (frequency deviants: 1.2 kHz, 50 ms; duration deviants: 1 kHz, 100 ms). Tones occurred every 330 ms. In total, there were 675 standards (82%), 75 frequency deviants (9%), and 75 duration deviants (9%). All stimuli were presented randomly and delivered by a headphone. Subjects were asked to watch a silent natural movie and ignore tones.

Subjects were seated 65 cm from the display in a sound-attenuated chamber with dim illumination. Scalp electroencephalogram (EEG) was recorded from 64-channel surface electrodes mounted in an elastic cap (EasyCap, Brain Products Inc., Bavaria, Germany). The reference electrode was placed on the tip of the nose, and AFz was used as the ground. EEG signals were recorded using a Vision Recorder with a sampling rate of 1000 Hz and a 0.016–200 Hz bandpass acquisition filter (BrainAmp, Brain Products Inc., Bavaria, Germany). Impedance for each electrode was kept below 5 kΩ.

#### EEG preprocessing and components extraction

Data were preprocessed and analyzed offline using EEGLAB (Delorme and Makeig, 2004), and customized MATLAB scripts (MathWorks, Inc., Natick, MA, USA). EEG data were band-pass filtered between 0.1 and 40 Hz through a Hamming windowed finite impulse response (FIR) filter. A notch filter was used to minimize remaining 50 Hz line noise. The “bad electrodes” (i.e., those electrodes with a large drift longer than 1 min) were identified through visual inspection and were interpolated using a spherical spline method (Perrin et al., 1989), less than two electrodes were marked as bad for each subject on average in 24 out of 108 participants, and epochs with obvious artifacts were manually removed by visual inspection. EEG data were down-sampled to 250 Hz and re-referenced to the average of two mastoid electrodes (TP9 and TP10). The independent component analysis (ICA) was applied to remove artifacts caused by eye movements & blinks, electromyography (EMG), electrocardiography (ECG), and any non-physiological artifacts (Chaumon et al., 2015).

EEG data were segmented into epochs of −100 ms to 400 ms time locked to auditory stimulus onsets and baseline corrected using the 100 ms pre-stimulus period. Individual epochs with voltage exceeding ± 100 μV were excluded. Epochs were separated into two groups (standards and duration deviants) and averaged, respectively. Individual MMN waveforms were obtained by calculating the difference between waveforms evoked by duration deviants and standards. MMN was measured over midline frontocentral electrodes (Fz, FCz, and Cz). The individual peak latency of MMN was identified as the time point of the most negative voltage during a pre-defined window (150–250 ms) for each subject. MMN amplitudes were measured as the mean voltages within the ± 20 ms window around the individual peak latency.

#### Statistical analysis

Statistical analyses were performed using SPSS (version 22.0, SPSS Inc., Chicago, USA). Group differences in terms of MMN amplitude and latency were examined via a three-way repeated measures analysis of variance (ANOVA), with age (Young-old and Old, stratification by the mean value, 73) and longitudinal cognitive change caculated by total RBANS index scores (Decline and Stable, cut-off value: median value, 2 for Young-old and 1 for Old, subjects at the median value are categorised in the Stable group) as the between-subjects factors and electrodes (Fz, FCz and Cz) as the within-subjects factor. To further reveal the age effect on MMN peak amplitude, Young-old adults with cognitive decline were excluded from the sample due to the effect of cognitive decline on MMN peak amplitude in this population. The remaining subjects were grouped in a 5-year interval, and MMN amplitude was analyzed via a two-way repeated measures ANOVA, with age (60–90 grouped in 5-year interval, groups with a sample size of less than 10 (60–65 years and 85–90 years) are merged into adjacent groups, Four groups: 60–70, 70–75, 75–80, 80–90) as the between-subjects factor and electrodes (Fz, FCz, and Cz) as the within-subjects factor. Bonferroni corrections were used for *post hoc* tests and pairwise comparisons. In order to further elucidate the alterations in MMN amplitude with increasing age. The relationship between age and MMN amplitude within a 10-year interval was analyzed by using Pearson’s correlation.

## Results

### MMN indexes as predictors of cognitive change

To investigate whether the MMN index can be used as a predictor of cognitive decline in older adults. One hundred six participants were divided into 4 groups according to their age and one-year change of RBANS scores: young-old adults with cognitive decline (Y-D, *n* = 27), young-old adults with cognitive stable (Y-S, *n* = 31), older adults with cognitive decline (O-D, *n* = 23) and older adults with cognitive stable (O-S, *n* = 25). Demographic characteristics and neuropsychological scores are described in Table 1. There were no significant differences between the four groups in the demographic data (gender, years of education), SAS, and SDS scores.

**Table 1:** Demographic characteristics and cognitive measures of four groups with different cognitive change stratified by age.

Parameter	Decline^a^ (*n* = 27)	Stable^b^ (*n* = 31)	Decline^c^ (*n* = 23)	Stable^d^ (*n* = 25)	F/Chi-square	*P*
	Young-old		Old			
Age, year, Mean (SD)	67.3 (5.2)	67.2 (3.7)	80.3 (3.9)	79.3 (4.4)	74.37	<0.0001**
Education, year, Mean (SD)	9.0 (4.3)	10.3 (2.5)	10.5 (3.9)	9.4 (4.0)	0.90	0.45
Gender, n (male/female)	8/19	9/22	9/14	11/14	1.89	0.60
SAS	38.3 (6.7)	34.9 (7.0)	39.0 (6.0)	35.5 (6.9)	2.32	0.08
SDS	39.0 (10.1)	38.1 (9.0)	39.0 (9.3)	36.0 (9.3)	0.54	0.66
MMSE	28.3 (2.3)	28.1 (1.7)	26.0 (3.1)	27.3 (2.6)	3.21	0.03*
RBANS index scores						
Working memory	86.8 (16.2)	83.6 (15.2)	80.6 (19.1)	75.6 (19.9)	1.92	0.13
Visuospatial	99.4 (12.7)	96.0 (13.0)	93.6 (15.9)	91.6 (13.1)	1.59	0.20
Language	97.0 (10.4)	97.2 (8.6)	92.8 (12.3)	93.8 (6.3)	1.41	0.24
Attention	96.9 (18.3)	103.9 (12.5)	93.5 (16.3)	90.8 (16.7)	3.55	0.028*
Delayed memory	95.4 (16.2)	95.1 (3.4)	83.7 (21.9)	86.8 (20.5)	2.51	0.07
Total	94.0 (15.4)	92.5 (10.0)	85.5 (15.0)	82.9 (15.6)	3.88	0.01*
1 year change	−3.7 (4.8)	10.5 (6.2)	−8.7 (7.0)	10.4 (7.0)	63.03	<0.0001**

SAS, Self-rating anxiety scale; SDS, Self-rating depression scale; RBANS, Repeatable Battery for the Assessment of Neuropsychological.

*Post hoc* test: Age: a, b < c, d; MMSE: a, b > c; RBANS: Attention: b > c, b > d; Total: a > c, a > d, b > d.

*p < 0.05, **p < 0.01.

**Figure 1:**
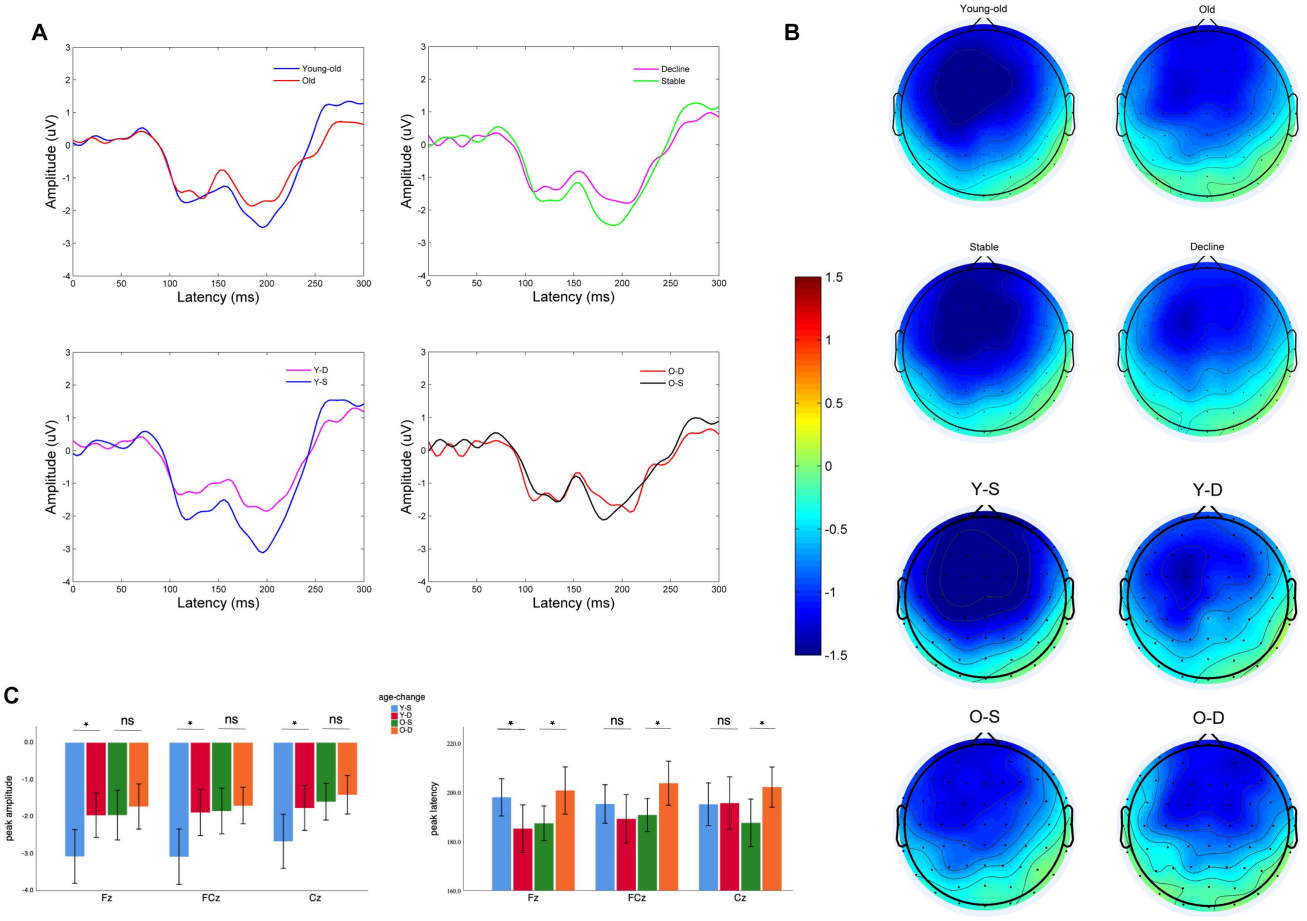
Electroencephalographic characteristics of older adults with different cognitive changes and ages: **(A)** MMN wave at Fz of two different groups, pairwise comparison: Young-old vs. Old, Cognitive decline vs. Cognitive stability, Young-old adults with cognitive decline (Y-D) vs. Young-old adults with cognitive stability (Y-S), Old adults with cognitive decline (O-D) vs. Old adults with cognitive stability (O-S). **(B)** Topography between 150 and 250 ms of two different groups, pairwise comparison: Young-old vs. Old, Cognitive decline vs. Cognitive stable, Y-D vs. Y-S, O-D vs. O-S. **(C)** Comparison of MMN peak amplitude and latency at different electrodes between four groups (Y-S, Y-D, O-S, and O-D). *p < 0.05, ns: Differences are not significant.

For the MMN amplitude, we found a significant main effect of age at midline frontal-central electrodes (*F* = 5.06, *p* = 0.027). Old adults have lower MMN amplitude than young-old adults at midline frontal-central electrodes (Supplementary Figure 1A: Old vs. Young-old: −1.71 ± 1.31 μV vs. −2.44 ± 1.83 μV, *p* = 0.027), between differences are significant at all three electrodes. Also, there was a significant main effect of cognitive change at midline frontal-central electrodes (*F* = 4.14, *p* = 0.044). Old adults with cognitive decline show lower MMN peak amplitude than old adults with cognitive stability (Figures 1A,B). But pairwise comparison shows that the difference is only observed in young-old adults (Figure 1C: −1.87 ± 1.48 vs. −2.94 ± 1.98 μV, *p* < 0.01) while it vanishes in older adults (Figure 1C: −1.61 ± 1.23 vs. −1.80 ± 1.41 μV, *p* = 0.68). We did not find an interaction between age and cognitive change (*F* = 1.96, *p* = 0.16) on MMN peak amplitude at the electrodes.

For the MMN peak latency, we did not find significant main effects of age or cognitive change at midline frontal-central electrodes (Supplementary Figures 1A,B: age: *F* = 0.39, *p* = 0.54; cognitive change: *F* = 1.02, *p* = 0.32), but there was a significant interaction between age and cognitive change at midline frontal-central electrodes (*F* = 7.04, *p* = 0.009). Young-old adults with cognitive decline show earlier MMN latency (Figure 1C: Decline vs. Stable: 190.0 ± 23.5 vs. 196.1 ± 20.3 ms, *p* = 0.015) when Older adults with cognitive decline show delayed MMN latency (Figure 1C: Decline vs. Stable: 202.1 ± 14.9 vs. 188.5 ± 14.8 ms, *p* = 0.225). Pairwise comparisons in Young-old adults is significant at Fz (*p* = 0.025). Pairwise comparisons in Older adults are significant at Fz (*p* = 0.033), FCz (*p* = 0.037), Cz (*p* = 0.035). Note that the cognitive performance of our sample of young-old adults was better than that of older adults. To exclude the effect of cognitive performance on MMN indexes, we further analyzed baseline cognitive performance as another between-subjects factor and found no significant main effect of cognitive performance or any interaction between cognitive performance and age or cognitive change on MMN indexes at midline electrodes, either in terms of latency or peak amplitude (Supplementary Table 1; Supplementary Figures 1C,D).

### Age effect on MMN peak amplitude

Seventy nine participants were divided into 4 groups according to their age at 5-year intervals, groups with a sample size of less than 10 (60–65 years and 85–90 years) are merged into adjacent groups. Demographic characteristics and neuropsychological scores are described in Table 2. There were no significant differences in the demographic data (gender, years of education), SAS, and SDS scores.

**Table 2:** Demographic characteristics and cognitive measures for older adults of different ages at 5-year intervals.

Age, year, Range	60–70^a#^ (*n* = 21)	70–75^b^ (*n* = 18)	75–80^c^ (*n* = 14)	80–90^d#^ (*n* = 26)	F/Chi-square	*P*
Education, year, Mean (SD)	10.4 (2.7)	10.1 (2.7)	10.8 (4.5)	9.3 (3.9)	0.67	0.57
Gender, n (male/female)	8/13	4/14	8/6	9/17	4.21	0.24
SAS	34.5 (6.7)	35.9 (7.4)	37.5 (7.6)	37.4 (7.6)	0.83	0.48
SDS	37.8 (9.4)	39.3 (10.1)	35.6 (8.0)	37.6 (9.1)	0.38	0.77
MMSE	28.2 (1.6)	27.9 (2.1)	28.2 (2.4)	25.6 (2.8)	5.28	0.004**
RBANS index scores						
Working memory	86.2 (14.9)	79.8 (16.9)	78.5 (12.4)	76.5 (22.8)	1.36	0.27
Visuospatial	98.4 (12.7)	92.6 (12.5)	93.9 (11.9)	91.2 (16.3)	1.12	0.35
Language	99.0 (8.8)	96.3 (7.0)	92.4 (7.1)	91.9 (11.1)	2.90	0.04*
Attention	105.1 (10.5)	101.3 (13.9)	96.5 (13.9)	86.9 (17.4)	6.60	0.001**
Delayed memory	97.3 (13.7)	92.1 (13.9)	91.6 (22.3)	79.2 (20.2)	4.28	0.01*
Total	94.9 (7.7)	89.5 (12.3)	87.4 (11.6)	80.0 (16.8)	5.85	0.002**

SAS, Self-rating anxiety scale; SDS, Self-rating depression scale; RBANS, Repeatable Battery for the Assessment of Neuropsychological.

*Post hoc* test: MMSE: a, b, c > d; RBANS: Language: a > c, a > d; Attention: a > d, b > d; Delayed memory: a > d; Total: a > d.

*p < 0.05, **p < 0.01.

^#^Groups with a sample size of less than 10 (60–65 years and 85–90 years) are merged into adjacent group.

**Figure 2:**
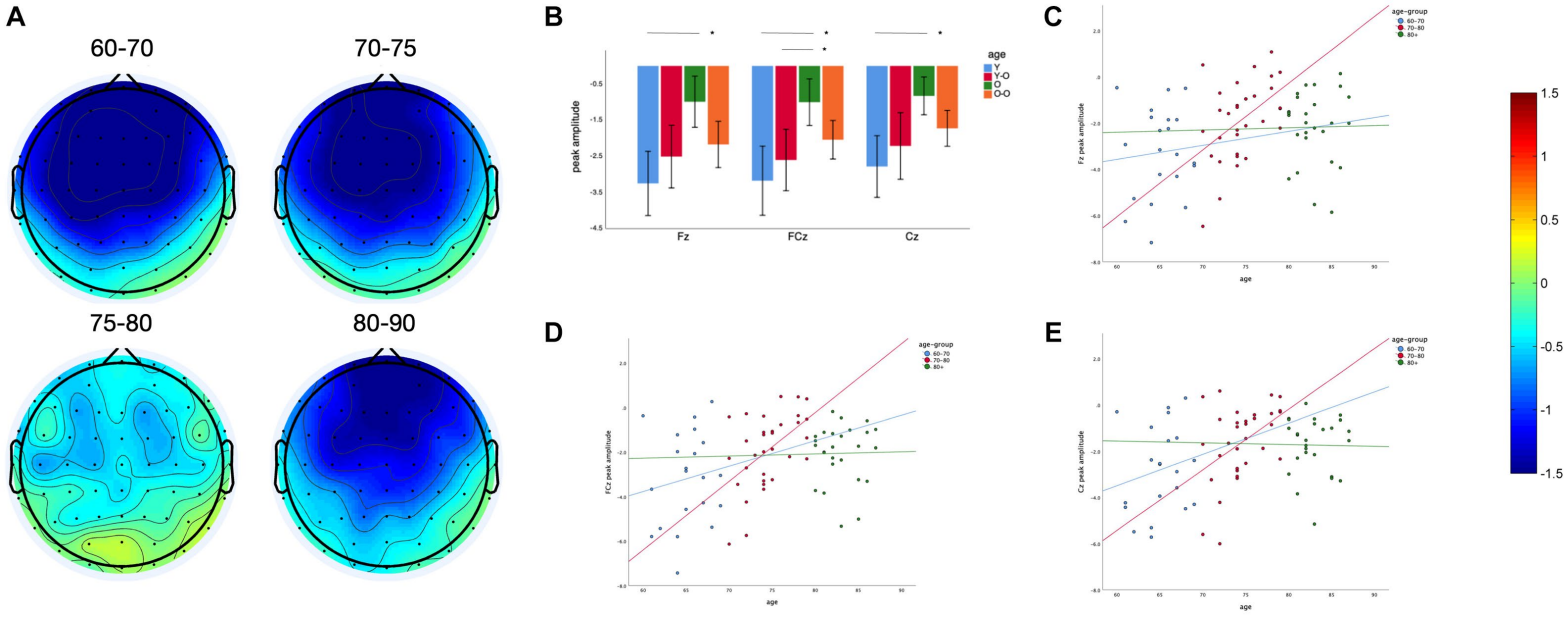
Age effect on MMN peak amplitude: **(A)** Topography between 150 and 250 ms of four different age groups. **(B)** Comparison of MMN peak amplitude at different electrodes between four groups. **(C)** Linear regression of age and MMN peak amplitude in three age groups with an interval of 10 years at Fz. **(D)** Linear regression of age and MMN peak amplitude in three age groups with an interval of 10 years at FCz. **(E)** Linear regression of age and MMN peak amplitude in three age groups with an interval of 10 years at Cz. *p < 0.05, ns: Differences are not significant.

We found a significant main effect of age on MMN amplitude at midline frontal-central electrodes (Fz, FCz and Cz, *F* = 5.41, *p* = 0.002). The older adults aged 75–80 years have a lower MMN amplitude than the older adults aged 60–70 and 70–75 years (Figures 2A,B). The difference between the 75–80 years and 60–70 years groups was significant at Fz (*p* = 0.001), FCz (*p* = 0.001), and Cz (*p* = 0.003). The difference between the 75–80 years and 70–75 years groups was significant at FCz (*p* = 0.043). For further correlation analysis, the sample was divided into 3 groups at 10-year intervals. Linear regression analysis showed that the MMN peak amplitude at three electrodes decreased with age in older adults within the 70–80 years age group (Fz: *p* = 0.006, Adjusted *R*^2^ = 0.20, FCz: *p* = 0.001, Adjusted *R*^2^ = 0.24, Cz: *p* = 0.01, Adjusted *R*^2^ = 0.17), with a slight but non-significant decreasing trend within the 60–70 years age group (Fz: *p* = 0.73, FCz: *p* = 0.54, Cz: *p* = 0.42; Figure 2D), and no similar trend was observed in older adults over 80 years of age (Fz: *p* = 0.95, FCz: *p* = 0.94, Cz: *p* = 0.95; Figures 2C–E).

## Discussion

Cohort studies have confirmed that hearing impairment (self-reported or behavioral measures) is associated with cognitive decline or the occurrence of dementia several years later (Maharani et al., 2018, 2020; Hwang et al., 2022). It has also been shown that central auditory processing as measured by binaural integration could identify older adults with different cognitive performance (Wang et al., 2022). There are several possible mechanisms for the relationship between hearing impairment and cognitive impairment: common pathology, impoverished input, occupation of cognitive resources and function-pathology interaction (Griffiths et al., 2020). A recent review highlighted the interaction of auditory processing with the hippocampus and the possible outcome on cognition (Billig et al., 2022). Moderate noise exposure in rats specifically impaired neuroplasticity in the hippocampus and cerebral cortex which led to deleterious cognitive outcomes, and a study in gorillas found that a deficit of auditory processes was associated with damage in the integrity of white matter in hippocampus-related structures, leading to extensive cognitive compromise (Gray et al., 2020; Zhang et al., 2021). In general, the MMN is composed of overlapping contributions from auditory and frontal-cortex processes, interestingly, recent animal studies have identified a hippocampal origin for MMN (Yi et al., 2022). Thus, MMN induced by sound variant detection reflects the brain’s auditory information processing, involving fine changes in cognitive processes such as perception, attention and memory, which can predict cognitive decline. Our results also support the global impact of auditory processing in the brain that auditory deficit indexed by MMN of older adults may predict global cognitive decline 1 year later. Furthermore, there are different modalities of MMN compromise in older adults in different age groups. Young-old adults with future cognitive decline showed a decrease in MMN peak amplitude, accompanied by a forward shift in MMN peak latency, whereas in older adults it showed a delay in MMN latency, and unchanged MMN peak amplitude. Different modalities of MMN variation reveal similar inefficient auditory processing. It is important that compared to cohort studies (Chaumon et al., 2015; Maharani et al., 2018, 2020), the present study shows that the MMN can predict more subtle cognitive decline by providing an indication of cognitive change after 1 year, not 5 years or more. In addition, the MMN, as a classical ERP component, has previously been used as an index of cognitive dysfunction in a variety of disorders, such as schizophrenia and autism (Naatanen et al., 2014). Our study found it’s a potential predictor of cognitive decline, together with the auditory oddball paradigm, which does not require a response from the subject, the MMN holds promise for the assessment of cognitive stability through portable devices and for timely and effective interventions for older adults at risk of cognitive decline.

The trend of decreasing MMN peak amplitude with increasing age in adulthood is well established, but the trajectory of the decline is unclear (Kiang et al., 2009). Previous studies have shown that older adults aged ~70 years exhibit reduced MMN amplitude compared to younger adults aged ~25 years (Cooper et al., 2006; Cheng et al., 2013; Nowak et al., 2016; Aghamolaei et al., 2018). MMN amplitude decreased significantly only with long inter-stimulus intervals (ISIs, 4000 ms) and remained stable or decreased insignificantly in the study with short ISIs (400 ms) in older adults aged ~70 years compared with middle-aged adults aged ~50 years (Ruzzoli et al., 2012; Correa-Jaraba et al., 2016). This phenomenon has been explained by the impaired maintenance of sensory memory traces in older adults, but the preserve of stimulus coding (Ruzzoli et al., 2012). More studies considered older adults as a homogeneous group without stratification. To our knowledge, there are no studies that have explored changes in indexes of MMN during aging in older adults. Our study suggests that MMN amplitude continues to decline in older adults as they age. Interestingly, older adults aged 70–80 years had an accelerated rate of decline in MMN amplitude. Older adults aged 75–80 years showed a significant decline in MMN amplitude compared to 70–75 years and 60–70 years, with no difference between older adults aged 70–75 years and aged 60–70 years in cognitively well-maintained older adults. Unexpectedly, older adults aged >80 years did not show a significant decrease in midline MMN peak amplitude. It is important to note that only older adults aged >80 years in our sample showed significantly inferior cognitive performance, and the rebounds in MMN peak amplitude may reflect the dissociation of frontal and temporal MMN at different cognitive levels previously reported (Ruzzoli et al., 2016). AD patients showed a frontal but not temporal MMN, whereas MCI patients showed a temporal, but not a frontal MMN, in comparison to healthy older adults, who showed MMN in both the frontal and temporal areas (Ruzzoli et al., 2016). Future studies are needed to establish more fine-grained effects of cognition and aging on MMN. Similar to a recent study of Alzheimer’s disease-related biomarker changes across life span, it showed that there were an accelerated decrease in hippocampal volume at the baseline age of 55–60 years, an accelerated decrease in cortical thickness and cognition at the baseline age of 65–70 years throughout the human lifespan. Accelerated declines in hippocampal volume and cognition continued after 70 years (Luo et al., 2022). We also found that auditory processing as indexed by MMN peak amplitude also existed an accelerated decrease at the baseline age of 70 years in our sample. It is important to establish the trajectory of biomarker changes across the lifespan to indicate effective and timely time points for intervention.

Finally, the results of the present study provide an interpretation regarding the inconsistent results of many previous studies that have attempted to develop MMN as a screening tool for cognitive performance, especially in mild cognitive impairment (MCI) patients. Current studies have not yielded a consensus on whether MMN peak amplitude is decreased and whether MMN peak latency is preceded or delayed in MCI patients (Mowszowski et al., 2012; Lindin et al., 2013; Ji et al., 2015; Ruzzoli et al., 2016). The effects of age and possibly cognitive decline overshadow the effects of mild cognitive impairment on MMN indexes, leading to the conclusion that MCI patients do not have compromised MMN compared to healthy older adults. It is important to note that MCI patients may indeed have auditory processing impairment (Wang et al., 2022), but in the presence of a significant effect of unknown factors, the MMN index becomes a less sensitive biomarker. As in our study, the sample was derived from community-dwelling cognitively well-maintained older adults. We did not find a significant effect of cognitive performance on the MMN index. The confounding factor of age is better controlled for, but given the unpredictability of future cognitive decline, perhaps the MMN is not a promising tool for screening cognitive performance.

Despite these interesting findings, there are several limitations to our study. First, the lack of young and middle-aged adult control in the sample prevented a comprehensive exploration of the trajectory of MMN index changes with age. Second, we did not examine auditory acuity by the pure-tone audiometry in older adults and were unable to explore age-related and cognitive-related changes in peripheral auditory processing and the relationship between different levels of auditory processing.

In summary, this is the first time we have found that the MMN indexes may predict cognitive decline at a 12-month follow-up. In addition, previous literature lacks information for the 70+ age group. Our data complements this lacuna. We found that MMN amplitudes will continue to decrease following the age of 70 years, and MMN amplitudes decrease at a more rapid pace in the 70–80 years decade as compared to the 60–70 decade. Further larger sample research is needed to validate its efficacy, as well as to develop wearable devices for assessing cognitive stability in older adults with the aim of timely intervention, to delineate the trajectory of auditory processing across the lifespan.

## Data availability statement

The original contributions presented in the study are included in the article/Supplementary material, further inquiries can be directed to the corresponding authors.

## Ethics statement

The studies involving humans were approved by the Ethics Committee in Shanghai Mental Health Center. The studies were conducted in accordance with the local legislation and institutional requirements. The participants provided their written informed consent to participate in this study.

## Author contributions

JY, XT, CL, and HD: study conception. XT: participants recruitment. XT and JY: experiments. JY: drafting of the manuscript. SL, LJ, KW, XC, LW, and JW: a critical review of the manuscript. All authors contributed to the article and approved the submitted version.

## Funding

This work was supported by National Key Research and Development Program of China (grant number 2022YFC3600600), Shanghai Municipal Commission of Health and Family Planning (grant number 2020YJZX0105), Shanghai Clinical Research Center for Mental Health (grant number 19MC1911100), Key Program of Clinical Research Center in Shanghai Mental Health Center (grant number CRC2017ZD01), and National Natural Science Foundation of China (grant number 81901400).

## Conflict of interest

The authors declare that the research was conducted in the absence of any commercial or financial relationships that could be construed as a potential conflict of interest.

## Publisher’s note

All claims expressed in this article are solely those of the authors and do not necessarily represent those of their affiliated organizations, or those of the publisher, the editors and the reviewers. Any product that may be evaluated in this article, or claim that may be made by its manufacturer, is not guaranteed or endorsed by the publisher.
