# Supplementary material for: Altered auditory processes pattern predicts cognitive decline in older adults: different modalities with aging
Source: Front Aging Neurosci. 2023 Sep 6;15:1230939. doi: 10.3389/fnagi.2023.1230939 (PMC10510405; doi:10.3389/fnagi.2023.1230939)
Supplement: Supplementary file 1 [file Data_Sheet_1.PDF]

Altered auditory processes pattern predicts cognitive decline in older adults: different modalities with aging

Junjie Yang BM[1#], Xiaochen Tang MS [1#], Shaohui Lin MM[1], Lijuan Jiang MS[1], Kai Wei PhD[1], Xinyi Cao PhD[1], Lingshan Wan[4], Jijun Wang PhD[1,2,3], Hansheng Ding MD[4*], Chunbo Li MD[1,2,3*]

[1]Shanghai Key Laboratory of Psychotic Disorders, Shanghai Mental Health Center, Shanghai Jiao Tong University School of Medicine, Shanghai, PR China;

[2]Institute of Psychology and Behavioral Science, Shanghai Jiao Tong University, Shanghai, PR China;

[3]CAS Center for Excellence in Brain Science and Intelligence Technology (CEBSIT), Chinese Academy of Science, Shanghai, PR China

[4]Shanghai Health Development Research Center, Shanghai Medical Information Center, Shanghai, PR China

Address for correspondence: Chunbo Li, MD, Shanghai Key Laboratory of Psychotic Disorders, Shanghai Mental Health Center, Shanghai Jiaotong University School of Medicine, 600 Wanping Nan Road, 200030 Shanghai, People's Republic of China. e-mail: licb@smhc.org.cn. Hansheng Ding, MD, Shanghai Health Development Research Center, Shanghai Medical Information Center, 602 Jianguo Xi Road, 200031 Shanghai, People's Republic of China. e-mail: dinghansheng@hotmail.com.

Cognition effect on MMN indexes

We performed a three-way repeated measures ANOVA with cognitive performance(cut-off value: RBANS total index score 90) as the third between-subjects factor. There was not a significant main effect of cognitive performance or any interaction between cognitive performance and age or cognitive change on MMN indexes at midline electrodes, either in terms of latency or peak amplitude(**eTable 1, eFigure 1C,D**).

eTable 1. Statistical results of the three-way repeated measures ANOVA.

MMN indexes	Effect of between-subjects factors	F	p
peak amplitude	cog	0.02	0.88
	cog*age	0.07	0.79
	cog*change	0.17	0.69
	cog*age*change	0.77	0.38
peak latency	cog	0.09	0.77
	cog*age	0.21	0.65
	cog*change	1.28	0.26
	cog*age*change	0.17	0.68

Notes: cog: cognitive performance at baseline. change: cognitive change after 1-year follow-up.

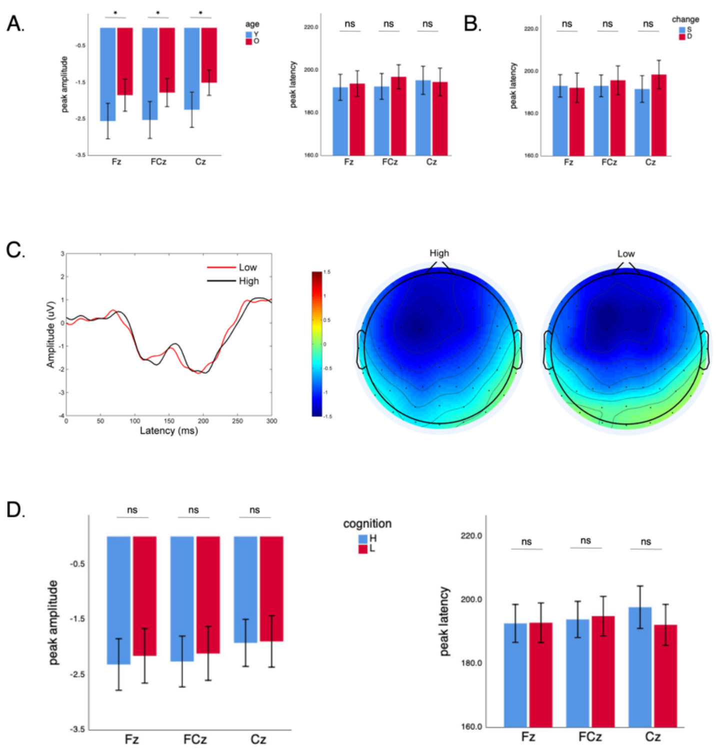

eFigure 1. Effect of age, cognitive performance and cognitive change on MMN indexes at Fz, FCz and Cz: A. Comparison of MMN peak amplitude and latency at different electrodes between different age groups. Topography between 150-250ms of our different age groups. **B.** Comparison of MMN peak latency at different electrodes between different cognitive change groups. **C.** MMN wave and topography between 150-250ms of two different cognition groups. **D.** Comparison of MMN peak amplitude and latency at different electrodes between different cognition groups. * : $p<0.05$, ns: Differences are not significant.
